# Multiscale Source Apportionment of Heavy Metals in Mining-Affected Farmland Soils Using PCA-PMF Modeling

**DOI:** 10.3390/toxics14070579

**Published:** 2026-06-30

**Authors:** Xiao-Zhou Deng, Yong-Hong Ma, Wen-Ying Wu, Zhi-Gang Peng, Zhi-Hao Zhao, Kun Gao, Jia-Jia Guo, Wei Chen

**Affiliations:** 1 Changsha General Survey of Natural Resources Center, Changsha 410600, China; dxzcs123@163.com (X.-Z.D.); masipoasf@163.com (Y.-H.M.); 122407@aqnu.edu.cn (W.-Y.W.); zgdzdcjcszxpzg@163.com (Z.-G.P.); kkkkkayn@163.com (Z.-H.Z.); gaokun720@126.com (K.G.); 2 Huangshan Observation and Research Station for Land-Water Resources, Huangshan 245400, China; 3 Xiaogan Observation and Research Station for Natural Resources, Xiaogan 432000, China; 4 Key Laboratory of Biodiversity Conservation and Characteristic Resource Utilization in Southwest Anhui, School of Life Sciences and Food Engineering, Anqing Normal University, Anqing 246133, China; 5 Changsha Water Industry Group Co., Ltd., Changsha 410015, China; 15084756208@163.com

**Keywords:** heavy metals, potential ecological risk, source identification, soil, spatial distribution

## Abstract

Polymetallic mining severely disrupts farmland soil ecosystems, yet the vertical migration of heavy metals, interlayer pollution disparities between topsoil and deep soil, and quantitative source apportionment of composite pollutants remain poorly understood in mining–agricultural overlapping zones. Two core hypotheses were accordingly proposed: mining-derived heavy metals can migrate downward and accumulate in deep soil layers, and the coupling of geostatistical analysis and receptor modeling enables reliable differentiation between geogenic and anthropogenic pollution sources. To test these hypotheses, 512 topsoil and 148 deep soil samples were collected from the Fenghuang Mining Area for quantification of eight metals and metalloids (including As). Geostatistical approaches, the single pollution index (Pi), and Nemerow comprehensive pollution index (P_N_) were utilized to characterize spatial heterogeneity and evaluate pollution severity, while a coupled PCA–PMF receptor model was adopted for quantitative source identification; vertical comparisons of element concentrations across soil profiles further validated the robustness of source apportionment outputs. The results revealed extensive heavy metal enrichment in both soil layers, with only topsoil Cd exceeding China’s risk screening value for agricultural land. Hg exhibited pronounced spatial variability and prominent anthropogenic fingerprints, and all target metals displayed consistent spatial distribution patterns along vertical soil profiles. Four distinct pollution sources were discriminated: geogenic sources dominating Cu, Zn, Cr, and Ni accumulation, mining-industrial emissions as the major contributor to Hg pollution, mixed industrial–agricultural inputs governing As and Pb enrichment, and traffic activities serving as the primary Cd source. Cd was identified as the priority pollutant threatening local farmland security. Confirmed downward percolation of anthropogenic metals creates persistent latent ecological risks across the study area, where mining and industrial discharges represent the dominant anthropogenic pollution inputs. This work systematically elucidates the geochemical signatures, vertical migration pathways, and quantitative source contributions of heavy metals in mining-disturbed farmlands, delivering solid scientific support for targeted source control, tiered risk management, and soil ecological remediation within the Fenghuang Mining Area. Moreover, the multi-method integrated analytical framework developed herein provides transferable guidance for heavy metal pollution mitigation in global polymetallic mining–agricultural regions with analogous geological and industrial backgrounds.

## 1. Introduction

This study aims to explore the effects of anthropogenic activities on soil heavy metal enrichment and clarify the impacts of vertical migration of soil background heavy metals on farmland soil quality. Soil is a significant resource for human survival and development, but with the intensification of industrialization and urbanization, multitudinous production and living activities have caused serious damage to the original properties of soil, among which heavy metal pollution is particularly prominent and has become an important global environmental problem [1]. Heavy metal pollutants are difficult to remove from soils, water, and living organisms, and can persist in the environment, accumulate, and cause long-lasting consequences. At the same time, they can accumulate step by step in organisms through the food chain, resulting in high concentrations in organisms far higher than their environmental concentrations. Additionally, most metals and metalloids are toxic to organisms, which can affect physiological functions, interfere with metabolic processes, and even lead to health problems such as gene mutations and cancer through the food chain [2], skin contact, and other means [3].

Mining activities constitute a typical anthropogenic source of heavy metal enrichment in regional soils. During mineral exploitation, tailings accumulation, open waste rock stacking, and uncontrolled discharge of mine wastewater, heavy metal-laden dust, residues, and leachate continuously spread to surrounding farmlands, altering native soil physicochemical properties, resulting in widespread heavy metal over-standard pollution and posing long-term risks to farmland ecology and agricultural product safety.

Pollution with heavy metals in southwestern and central-southern regions of China is severe, characterized by widespread exceedances of standards and significant challenges in investigation and analysis. Although conventional soil heavy metal survey methods deliver reliable detection precision, they face difficulties in subsequent pollutant source tracing and involve a lengthy tracing cycle. Therefore, to protect soil resources, we urgently require methods capable of rapidly assessing the severity of soil heavy metal pollution and elucidating its pollution sources.

Currently, field sampling combined with computer inversion modeling is the primary method for analyzing soil heavy metal pollution. The computer inversion simulation methods for source analysis of soil metals and metalloids can be primarily categorized into two types: qualitative source identification and quantitative source apportionment [4]. Principal Component Analysis (PCA) and Positive Matrix Factorization (PMF) model analysis methods are currently the mainstream qualitative analysis methods, which can effectively reduce the cost and workload of soil remediation and quantitatively elucidate the sources of heavy metal pollution in soil [5,6]. However, in practical applications, PCA, as a multivariate statistical analysis method, cannot directly quantify the specific contributions of different pollution sources [7]. Similarly, the results of the PMF model are sensitive to data samples; several basic assumptions need to be met, and when these conditions are not met, PMF may not be successful in the source allocation of soil metals and metalloids [8,9]. One key assumption, for instance, is that the total pollutant content equals the sum of contributions from all individual sources. In reality, localized human activities (e.g., traffic emissions, agriculture, industry) may not fully represent the sources of metals and metalloids across an entire region [10]. These limitations highlight remaining shortcomings in both analytical approaches.

PCA possesses the advantage of qualitatively identifying and classifying pollution sources [11], but it cannot directly quantify the specific contributions of different pollution sources. PMF does not require source component spectrum data, calculates factor distribution, quantifies the contributions of each factor, and can handle missing data and data with large errors [12,13,14]. Therefore, in this research principal component analysis (PCA) and positive matrix factorization (PMF) were employed to explore the interrelationships between each heavy metal, and based on the mutual support of PCA and PMF prediction results, determines the sources of metals and metalloids by combining the distribution characteristics of elements in the study area and the heavy metal content in deep soil, in order to improve the accuracy of the research.

The main objectives of this study were to (1) analyze the spatial distribution characteristics of metals and metalloids in soil in Fenghuang County, China, (2) identify and analysis potential sources of metals and metalloids through PCA and PMF model, (3) to combine the content of metals and metalloids in deep soil and the distribution of mining sites in the study area to verify the results of PCA and PMF models, providing effective suggestions for the prevention and control of heavy metal pollution.

## 2. Materials and Methods

### 2.1. Study Area

The study area is located in Hunan Province, China. On the western edge of Hunan Province (109°18′~109°48′ E, 27°44′~28°28′ N), including the entire Fenghuang County and part of Huayuan County, it is 50 km wide from east to west and has a total land area of 2044 km^2^.

The study area is abundant in mineral resources, with 35 exploitable mineral deposits identified so far, such as natural diamonds, mercury, lead-zinc ore, cement limestone, anthracite, stone coal, marble, silica, and antimony; notably, its mercury reserves rank fourth nationwide. Long-term mining and ore processing activities have triggered severe soil heavy metal contamination, and field surveys reveal prominent over-standard levels of Cd, Hg, As, and other metals and metalloids in local soils, which severely impair regional agricultural production and soil quality. Given the rich mineral reserves coupled with prominent environmental pollution risks, this study takes the entire Fenghuang County and part of Huayuan County as the research scope.

### 2.2. Sample Collection

Soil sampling was implemented in strict accordance with the layout and collection methods of soil geochemical samples specified in the Specification for Multi-purpose Regional Geochemical Survey (1:250,000) (DZ/T 0258—2016).

**Figure 1:**
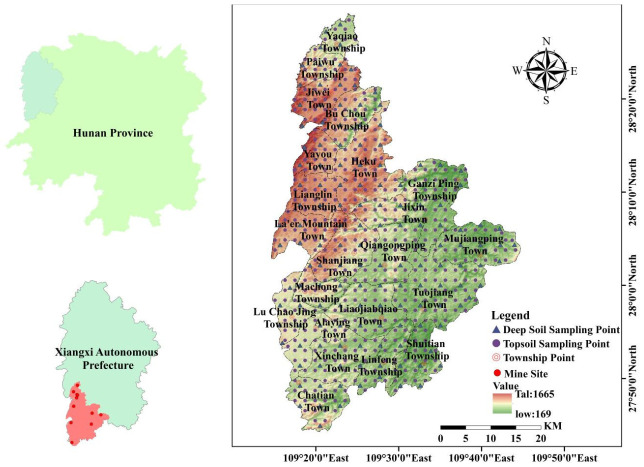
Sampling point distribution in the study area.

Field sampling yielded 512 topsoil samples from the 0–20 cm depth at a density of 1 sample/km^2^. Four equal subsamples were mixed to form a composite sample to reduce random errors. All raw samples had an initial weight above 1 kg, and samples collected within each 4 km^2^ unit were combined for subsequent analysis. A total of 148 deep soil samples were also obtained from the 150–180 cm layer at a density of 1 sample/4 km^2^ (Figure 1). All soil samples were then air-dried under ventilated conditions, followed by the removal of weeds, roots, gravel, bricks, and organic residues. The processed samples were manually crushed, thoroughly homogenized, sieved using a 100-mesh sieve, and sealed in bags. Finally, these pretreated specimens were sent to the Wuhan Mineral Resources Supervision and Testing Center, Ministry of Land and Resources, for chemical testing.

### 2.3. Sample Analysis

The Cr content was directly measured by X-ray fluorescence spectroscopy [15]; the As and Hg content was measured by atomic fluorescence spectroscopy [16]; Pb, Cu, Zn, and Cd were determined by inductively coupled plasma mass spectrometry [17]; and the Ni content was determined by inductively coupled plasma atomic emission spectroscopy (ICP-AES). The analytical methods and detection limits of all elements are detailed in Table 1.

### 2.4. Evaluation Methods for Metals and Metalloids

#### 2.4.1. Pollution Assessment Methods

The concentration of metals and metalloids in the topsoil and deep soil of the research area is based on the national environmental quality agricultural land soil pollution risk control standard (trial) (GB 15618-2018) [18]. The background values for Cr, Cd, Hg, As, Cu, Pb, Zn, and Ni are 71.4, 0.13, 0.12, 15.7, 27.3, 29.7, 94.4, and 31 mg/kg, respectively. The metals and metalloids in 8 types of soils in the study area were evaluated using the pollution index [19] and the Nemerow comprehensive pollution index [20]. Among them, the pollution index evaluates the main pollution factors and pollution status of the sample. The Nemero Comprehensive Pollution Index comprehensively reflects the overall pollution status of environmental media, and the expressions for the two methods are as follows:

(1) $$P_{i}\;=\;\frac{C_{i}}{S_{i}}$$

where P_i_ is the single factor pollution index of pollutant i in soil; C_i_ is the measured content of pollutant i in the soil (mg/kg); and S_i_ is the evaluation standard for pollutant i. This study used Soil geochemical data of China (partIII) as background values.

(2) $$P_{N}=\sqrt{\frac{{\left(\frac{C_{i}}{S_{i}}\right)}_{\max}^{2}+{\left(\frac{C_{i}}{S_{i}}\right)}_{\mathrm{ave}}^{2}}{2}}$$

P_N_ is the Nemerow integrated pollution index of soil heavy metal i, (Ci/Si)max is the maximum value of the single pollution index of metals and metalloids in soil; (Ci/Si)ave is the average value of the soil heavy metal single pollution index. P_N_ ≤ 0.7, Soil cleaning; 0.7 < P_N_ ≤ 1, warning threshold; 1 < P_N_ ≤ 2, mild pollution, 2 < P_N_ ≤ 3, moderate pollution, P_N_ > 3, severe soil pollution.

#### 2.4.2. Potential Ecological Risk Assessment Methods

The potential ecological risk index was proposed by Swedish scholar Hakanson [21]. This index combines toxicology, environmental chemistry, and ecological effects to express the potential ecological risks of metals and metalloids through intuitive and interpretable quantitative values. It is currently the most commonly used method for evaluating the degree of soil heavy metal pollution and ecological risks [22,23,24], and its calculation formula is:

(3) $$\mathrm{RI}\;=\;\sum E_{r}^{\;i}=\sum T_{r}^{\;i}\;\times \;C_{f}^{\;i}=\sum T_{r}^{\;i}\times \frac{C_{i}}{C_{n}^{\;i}}$$

RI is the comprehensive potential ecological hazard index of metals and metalloids in a single soil sample; $E_{r}^{\;i}$ is the potential ecological hazard coefficient of pollutant i in the soil; Tri is the toxicity response coefficient of pollutant i in the soil; $C_{f}^{\;i}$ is the pollution parameter of pollutant i in the soil; Ci is the measured content of pollutant i in soil; $C_{n}^{\;i}$ is the background value of soil elements in Hunan Province. This study refers to the standardized toxicity impact coefficients of heavy metal elements proposed by Hakanson. The toxicity coefficients of metals and metalloids Hg, As, Cd, Cr, Pb, Cu, Zn, and Ni are 40, 10, 30, 2, 5, 5, 1, and 5, respectively [25]. The classification criteria of $E_{r}^{\;i}$ and RI are listed in Table 2.

#### 2.4.3. Source Apportionment

PCA projects the original data from high-dimensional space to low-dimensional space through linear transformation, transforming the original variables into a few unrelated principal component variables that can preserve as much variance information as possible, thereby achieving data dimensionality reduction. This method can use fewer representative factors to explain the main information of numerous variables and infer information about pollution sources [26].

PMF is an effective data analysis method proposed by Paatero and Tapper in 1993 for parsing hidden structures in complex datasets. It is widely used for identifying the sources of environmental pollutants, and its equation is as follows:

(4) $$X_{\mathrm{ij}}\;=\sum_{k=1}^{p}G_{\mathrm{ik}}F_{\mathrm{kj}}+E_{\mathrm{ij}}$$

(5) $$Q=\sum_{i=1}^{n}\sum_{j=1}^{m}{\left(\frac{E_{\mathrm{ij}}}{U_{\mathrm{ij}}}\right)}^{2}$$

X_ij_ is the concentration of j elements in the i-th sample, G_ik_ is the contribution of k pollution sources to the i-th sample, F_kj_ is the concentration of j elements in k pollution sources, p is the number of factors, E_ij_ represents residual, and U_ij_ represents uncertainty.

The PMF model runs using concentration data and uncertainty data. Uncertain data includes sampling errors and analysis errors [27]. When the concentration of an element is less than its detection limit (MDL), the calculation method for its uncertainty is:

(6) $$U_{\mathrm{nc}1}=\;\left(5/6\right)\;\times \;\mathrm{MDL}$$

When the element concentration is greater than the detection limit (MDL), there are two methods for calculating its uncertainty:

(7) $$U_{\mathrm{nc}1}=\sqrt{{\left(\theta +C\right)}^{2}+{\left(0.5\times \mathrm{MDL}\right)}^{2}}$$

(8) $$U_{\mathrm{nc}2}=0.1\;\times \;C+\frac{1}{3}\mathrm{MDL}$$

MDL is the detection limit of the method, C is the test concentration, and is the coefficient of variation of each element content. To improve the accuracy of the model, after multiple experiments and considering the sample concentration and instrument monitoring characteristics, the uncertainty calculation method for different elements in this study is as follows: Cu, As, Zn use Formula (7), and Hg, Cd, Ni, Pb, Cr use Formula (8) [28].

#### 2.4.4. Data Analysis

This study used IBM SPSS 26 and Excel 2013 software to perform descriptive statistics, correlation analysis, and principal component analysis (PCA) on the data. EPA PMF 5.0 software was used for traceability analysis, ArcGIS 10.7 software was used for ordinary Kriging interpolation of soil metals and metalloids to obtain spatial distribution maps, and Origin 2021 was used to draw relevant maps.

## 3. Results and Discussion

### 3.1. Descriptive Statistics of Soil Heavy Metal Concentrations

Analysis results of heavy metal characteristics in 512 topsoil samples from the study area are presented in Table 3. The topsoil in the study area exhibited slightly acidic properties with a mean pH of 6.17. Mean concentrations (mg/kg) of monitored metals and metalloids were quantified as: Cr (75.22), Cd (0.47), Hg (0.42), As (14.95), Cu (32.84), Pb (42.15), Zn (103.14), and Ni (36.02). These values represent 1.05, 3.70, 3.61, 0.95, 1.20, 1.42, 1.09, and 1.13 fold of Hunan background values, respectively, with all elements except as exceeding regional baselines. Reference was made to the soil pollution risk screening values for soils with 5.5 < pH ≤ 6.5 specified in the Soil Environmental Quality Risk Control Standard for Agricultural Land (Trial) (GB 15618-2018). Notably, cadmium (Cd) concentrations exceeded the national risk screening thresholds, implying potential soil contamination hazards in the study area.

The analytical results of the tested elements in 148 deep soil samples from the study area are presented in Table 4. Compared with topsoils, the average concentrations of six heavy metal elements (excluding Cd and Hg) in deep soils were higher than those in topsoils, and the pH of deep soils was also higher. A comparison of the data presented in Table 3 and Table 4 reveals that both soil types exhibit high coefficients of variation for Cd, Hg, and As, suggesting significant spatial heterogeneity of these three metals and metalloids [29].

### 3.2. Spatial Distribution Characteristics of Metals and Metalloids

**Figure 2:**
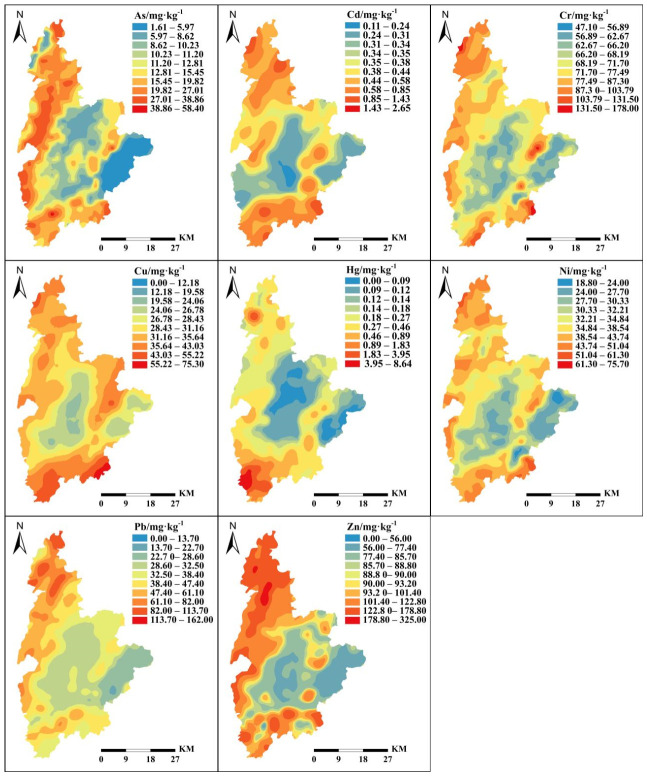
Characteristic distribution of heavy metal concentrations in topsoil (0–20 cm).

The spatial distribution characteristics of eight metals and metalloids in the topsoil of the study area are illustrated in Figure 2. The low-concentration zones of all eight metals and metalloids are distributed in the central and eastern parts of the study area. The high-value zones of As, Cd, Pb, and Zn are primarily concentrated in the northern part of the study area. Among these, As peaked at 58.4 mg/kg, Cd at 2.65 mg/kg, Zn at 325 mg/kg, and Pb at 162 mg/kg. High-value zones of Cu and Hg are mainly distributed in the southern part of the study area, with Cu reaching 75.3 mg/kg and Hg 8.64 mg/kg. In contrast, Ni and Cr exhibit relatively scattered distributions in their high-value zones.

**Figure 3:**
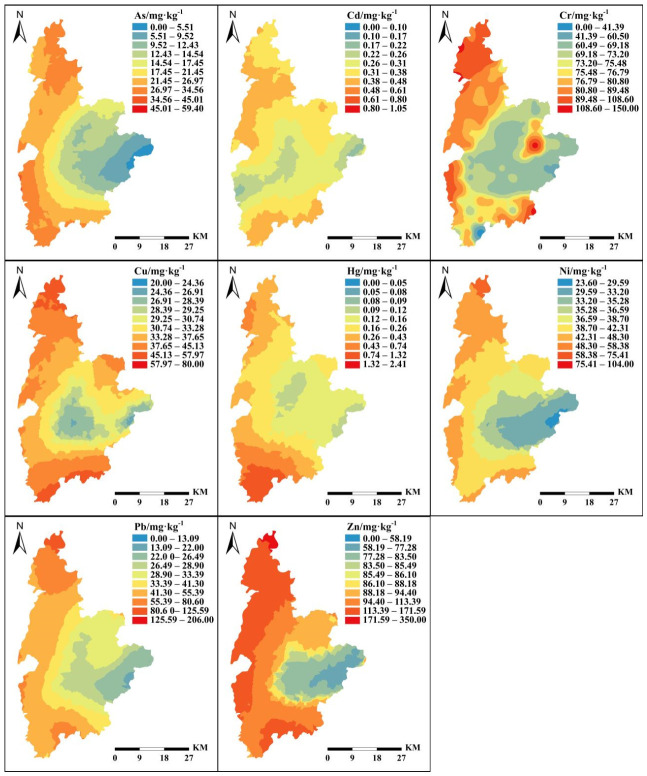
Spatial distribution characteristics of heavy metal concentrations in subsoil (150–180 cm).

Figure 3 presents the spatial distribution characteristics of heavy metals and metalloids in deep soil across the study area. The spatial distribution pattern of heavy metal concentrations in deep soil is generally consistent with that in topsoil, and low-concentration zones are mainly clustered in the central part of the study area. High-value zones of As, Cr, Cu, and Zn are distributed in both the southern and northern regions of the study area; high-value zones of Pb and Ni are primarily concentrated in the north, whereas Hg high-value zones are mostly distributed in the south. Combined with the distribution pattern of major mine sites shown in Figure 1, it can be found that the high-concentration zones of As, Cr, Cu, Zn, Pb, and Ni are highly spatially overlapped with the concentrated mining areas in the northern study area.

Mining activities in the northern mining belt, including ore excavation, tailings stockpiling, and waste rock weathering, continuously release heavy metal pollutants. Heavy metal-laden dust spreads with wind, and leachate formed by rainwater erosion migrates to surrounding farmlands through surface runoff and vertical soil infiltration, leading to significant heavy metal enrichment in the topsoil adjacent to mining areas.

### 3.3. Environmental Risk Assessment of Heavy Metal

Based on the evaluation criteria of the single-factor pollution index (Pi) and comprehensive pollution index (P_N_) for eight metals and metalloids in top-layer and deep-layer soils, data analysis was conducted on the proportion of pollution levels at each sampling point (Table 5). Topsoil Pi values indicate that most sampling points for the eight metals and metalloids fall within safe or slight pollution levels; Cu, Cr, Zn, and Ni exhibited no samples with severe pollution; a small number of the tested samples showed moderate pollution for Cu, Pb, Zn, and Ni; severe pollution samples were observed for Cd, Hg, As, and Pb. According to P_N_ can be seen that 46.48% of samples exhibited slight pollution, 14.65% showed moderate pollution, 16.99% registered severe pollution, and 21.88% remained unpolluted.

In the subtopsoil layer, with the exception of Cd and Hg, the majority of sampling points for other metals and metalloids exhibited safe or low contamination levels. While Cu and Cr showed no samples with heavy contamination, all other heavy metal elements documented samples with severe contamination. Based on the comprehensive pollution index (P_N_), the subtopsoil analysis reveals that merely 1.35% of samples remain uncontaminated, 41.89% exhibit slight pollution, 25% demonstrate moderate pollution, and 31.76% show severe pollution. The combined moderate and severe pollution accounts for 56.76% of samples, indicating that anthropogenic impacts from topsoil have permeated deep soil layers.

This study evaluated the comprehensive pollution level of metals and metalloids in topsoils of the research area using the potential ecological hazard index method (Table 6). As deep soils are not exposed to atmospheric environments, their heavy metal contamination was not assessed. Most sampling sites exhibited low ecological risk, while a small proportion showed very high to extremely high risk, indicating potential ecological hazards across the region. Sites with high and extreme ecological risks are mainly distributed in farmlands surrounding mining areas, while moderate-risk sites are concentrated along major traffic routes. The risk classification data can support hierarchical management of local farmlands and targeted arrangement of soil pollution prevention and remediation work. Heavy metal accumulation in farmland soil will deteriorate soil quality, cause metal enrichment in crops, and further threaten agricultural product safety and human health via the food chain. Corresponding control measures are proposed according to risk grades: regular monitoring is sufficient for low-risk farmlands; source control and agricultural regulation are required for moderate-risk areas; and high-risk and extreme-risk farmlands need to adopt pollution blocking and soil remediation measures. In addition, the downward migration of pollutants from topsoil may continuously affect deep soil, posing long-term hidden risks to regional farmland security.

### 3.4. Source Analysis of Metals and Metalloids

#### 3.4.1. Correlation Analysis

**Figure 4:**
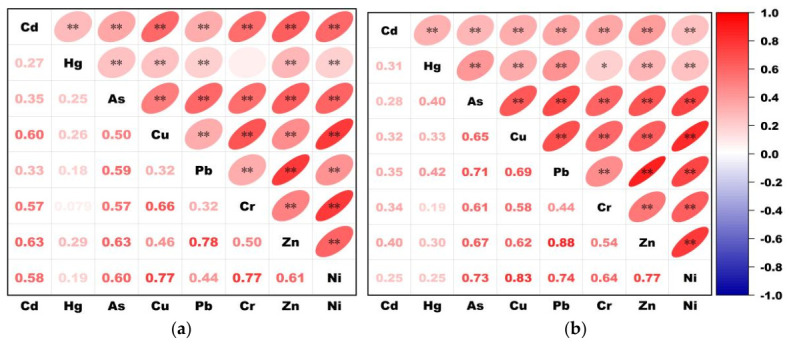
Correlation of metals and metalloids in topsoil (**a**) and deep soils (**b**). *: *p* < 0.05; **: *p* < 0.01.

To identify the sources of metals and metalloids, a correlation analysis was conducted on eight metals and metalloids in the topsoil and deep soils of the study area, where * in the figure indicates *p* ≤ 0.05 and ** indicates *p* ≤ 0.01. As shown in Figure 4a, except for Hg, the other seven heavy metal elements in topsoils exhibit significant positive correlations (*p* ≤ 0.01), indicating that these metals and metalloids may share the same sources and demonstrate strong companionship relationships [30].

In the deep soil layer, as shown in Figure 4b, except for the two elements Cd and Hg, the other six metals and metalloid elements showed significant positive correlations, with Pb and Zn having the highest correlation coefficient (0.88), which is similar to the topsoil.

The lack of significant correlation between Cd and Hg in both topsoil and deep soil is mainly attributed to their independent pollution sources, inconsistent spatial distribution, and different pathways of pollutant input. Cd originates primarily from traffic activities, while Hg is closely linked to mining and smelting activities. Their distinct geochemical characteristics and migration rules further weaken their correlation.

#### 3.4.2. PCA

To effectively identify the sources of metals and metalloids in the soil, this study employed SPSS software to conduct principal component analysis (PCA) on both topsoil and deep soil samples from the research area. The results are presented in Table 7. Three principal components were extracted for the metals and metalloids in the topsoil, with contribution rates of 52.50%, 14.11%, and 12.55%, respectively, achieving a cumulative variance contribution rate of 79.16%. In the first principal component, seven heavy metal elements—Ni, Zn, As, Cd, Cr, Pb, and Cu—exhibited significant loading values, with weight coefficients of 0.85, 0.83, 0.78, 0.77, 0.76, 0.71, and 0.71, respectively. This indicates a high likelihood of homologous origin for these seven metals and metalloids, excluding Hg. Several studies have found that chromium and nickel in agricultural soils primarily originate from parent materials [31], while cadmium, copper, and zinc stem from the excessive use of pesticides in agricultural activities. This suggests that Component 1 represents a mixed pollution source, potentially derived from agricultural pollution or natural background.

In the second principal component, the elements Pb and Zn have relatively high loadings, with weight coefficients of 0.62 and 0.47, respectively. The variance contribution rate of this principal component is 14.11%. Further supported by correlation analysis, it can be observed that lead and zinc exhibit a high correlation, suggesting a likely common origin. The study area is surrounded by numerous industrial and agricultural product processing factories, and industrial development has a significant impact on the soil content of Pb and Zn [32]. Therefore, principal component 2 is identified as a mixed source originating from both industrial and agricultural activities.

The third principal component has a contribution rate of 12.54%, with Hg demonstrating a markedly high weight coefficient of 0.99. The average detected Hg content in the topsoil of the study area is 0.42 mg/kg, which is 3.5 times higher than the background value for topsoils in Hunan Province (0.12 mg/kg), indicating significant Hg enrichment in the area. Furthermore, the coefficient of variation for Hg exceeds 100%, suggesting substantial influence from human activities. Studies have shown that Hg pollution in soil primarily originates from anthropogenic production activities such as mining and smelting. Considering the presence of historical mining sites in areas with high Hg concentrations within the study area, it is inferred that anthropogenic mining and smelting activities have led to the substantial accumulation of Hg in the soil.

For principal component 1 in the subtopsoil, the variance contribution rate is 45.76%. Elements such as Cu, Ni, As, Zn, Cr, Cd, and Hg exhibit relatively high positive loadings (0.45–0.86), indicating that these elements in the subtopsoil likely share a common origin, primarily controlled by the geological background. Principal component 2 has a variance contribution rate of 19.63%, with Pb and Zn showing significant positive loadings (0.79 and 0.62, respectively). This may reflect anthropogenic pollution inputs, and the results are consistent with the source analysis of topsoil pollution, suggesting that their common origin can be traced back to agricultural activities.

#### 3.4.3. PMF

**Figure 5:**
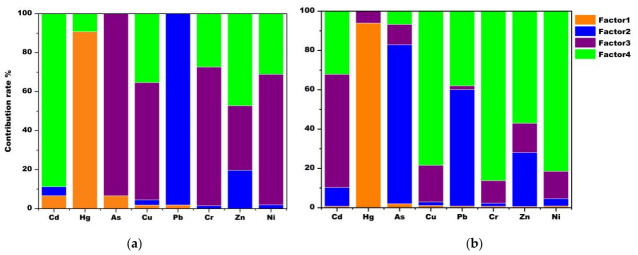
Factor analysis of metals and metalloids sources in topsoil (**a**) and deep soils (**b**) of the study area.

To further investigate and analyze the relationship and sources of metals and metalloids between the topsoil and deep soil in the study area, positive matrix factorization (PMF) was applied to analyze eight metals and metalloids in both topsoil and deep soil layers. The results are shown in Figure 5.

Figure 5a shows that Factor 1 contributed significantly to Hg in topsoil, with a contribution rate of 93.8%. The spatial distribution characteristics of Hg in the study area align with the locations of mining sites. Fenghuang County in Hunan Province hosts mercury deposits with a long history of exploration and mining. Additionally, the average Hg concentration in topsoil is notably higher than that in deep soil, indicating a substantial impact from mining activities on Hg content in the soil. Therefore, Factor 1 is identified as an anthropogenic source related to mining and industrial activities.

Factor 2 is primarily characterized by high loadings of As (80.9%), with minor contributions from Pb (59.3%) and Zn (27.4%). Fenghuang County’s industrial profile is dominated by agro-food processing and electronic manufacturing sectors. The accumulation of lead and arsenic in soil mainly stems from industrial atmospheric deposition [33], while the increased zinc levels are largely attributed to the application of animal manure and phosphate fertilizers [34]. Therefore, Factor 2 is identified as a mixed source derived from both industrial and agricultural activities.

Factor 3 exhibits a high contribution rate to Cd, at 57.6%. As shown in Table 3, the coefficient of variation for Cd is 64.59%, and its concentrated distribution area intersects with three main tourist routes traversed by national, provincial, and county highways. Previous research has linked Cd to transportation sources, as vehicle emissions—which can deposit via atmospheric sedimentation and dust migration—accumulate in soils and farmlands, leading to increased Cd concentrations. Concurrently, Cd high-value areas in the study region coincide with mining sites, where intensive vehicle transport and other human activities associated with mining operations occur [35]. In summary, Factor 3 is determined to originate from transportation sources.

Factor 4 shows high contribution rates to Cu, Zn, Cr, and Ni, at 78.4%, 57%, 86.2%, and 81.5%, respectively. The coefficients of variation for these four elements are relatively low: 26.75%, 34.61%, 19.56%, and 22.37%, suggesting minimal anthropogenic disturbance. A comparison of element concentrations between topsoil and deep soil layers reveals higher values in deep soil, indicating that these elements mainly derive from the local natural environment. Furthermore, the single-factor pollution index (Pi) for these four elements in topsoil is predominantly at safe or slightly polluted levels. Therefore, Factor 4 is classified as a natural source.

Figure 5b shows the four factors analyzed by the PMF model in subtopsoil, which are generally consistent with the elements represented by the factors in topsoil. Factor 1 is dominated by mercury (90.73%), Factor 2 by lead (98.14%), Factor 3 by arsenic (93.39%), copper (60.13%), chromium (71.13%), and nickel (66.98%), while Factor 4 is characterized by cadmium (88.77%). A comparison of the mean concentrations of metals and metalloids in topsoil and deep soils reveals that only mercury and cadmium show higher average concentrations in topsoil. Combined with PMF analysis, this suggests that mercury and cadmium may have persistent sources, while the other metals and metalloids likely originate from non-persistent sources or are controlled by geological backgrounds.

## 4. Discussion

### 4.1. Spatial Distribution and Vertical Migration of Metals and Metalloids

This study employed the single-factor index method, correlation analysis, principal component analysis (PCA), and positive matrix factorization (PMF) receptor model, combined with spatial distribution analysis, to systematically investigate the pollution level, spatial pattern, and source apportionment of metals and metalloids in surface and deep farmland soils of the Fenghuang Mining Area. The results indicated that, except for As, the concentrations of the other seven metals and metalloids were all higher than the soil background values of Hunan Province. Only Cd in topsoils exceeded the screening threshold specified in GB 15618-2018. This demonstrates that the study area was markedly disturbed by mining and human activities, with Cd identified as the primary over-standard pollutant in farmland soils.

Spatial distribution of metals and metalloids showed a high similarity between topsoil and deep soils. The high-concentration zones of all eight metals and metalloids were mainly distributed at the northern and southern ends as well as the western margin of the study area, while the central region was dominated by low-value zones. Such a spatial pattern implies evident vertical migration and diffusion of metals and metalloids, and vertical transport serves as the predominant migration pathway in this area. Moreover, the average contents of Cd and Hg in topsoils were remarkably higher than those in deep soils, showing prominent surface enrichment. By contrast, Cu, Zn, Cr, and Ni presented higher concentrations in deep soils with low variation coefficients, weak anthropogenic interference, and predominant control by regional geological background. This vertical difference provides solid stratigraphic and spatial evidence for the PCA-PMF source identification results. Meanwhile, it demonstrates that the spatial distribution and vertical migration of metals and metalloids are jointly regulated by regional geological conditions and human activities, including mining and transportation [36]. The contaminated soils in this area pose potential environmental risks to local farmlands [37].

### 4.2. Stratification Characteristics of Heavy Metal Pollution Risk

Pollution assessment revealed extensive heavy metal contamination risks in both topsoil and deep soils, with a high proportion of moderately and severely polluted sites. Anthropogenic heavy metal inputs from the surface have infiltrated downward into deep soils, showing an obvious downward diffusion trend. The single-factor pollution index indicated no severe pollution for Cu, Cr, Zn, and Ni, with their overall pollution risks remaining controllable. In comparison, severely polluted samples of Hg accounted for 21.72%. Combined with its high coefficient of variation, spatial overlap with mining sites, and model simulation, Hg was primarily derived from mining, smelting, and other industrial activities, acting as a key contributor to regional ecological risk [38,39]. Cadmium exhibited serious over-standard levels, and its high-value areas highly coincided with traffic networks and mining transport routes, suggesting Cd was mainly affected by traffic sources combined with mining transportation. As, Pb, and Zn were collectively influenced by industrial–agricultural emissions, agrochemical application, and atmospheric deposition, categorized as mixed industrial–agricultural sources. Cu, Zn, Cr, and Ni mainly originated from natural geological parent materials, with limited anthropogenic contribution. Pollutant concentrations and pollution levels are closely associated with their corresponding pollution sources. Long-term heavy metal accumulation has adverse effects on soil quality and crop safety of farmlands.

### 4.3. Heavy Metal Correlation and Source Differentiation

Correlation analysis showed that all metals and metalloids except Hg in topsoils had extremely significant positive pairwise correlations. In deep soils, significant correlations were observed for all elements except Cd and Hg. It is indicated that most metals and metalloids share homologous sources and paragenetic enrichment features, whereas Hg and Cd display obvious differentiation and are dominated by specific anthropogenic sources. The correlation differences between elements further verify their distinct pollution sources, which is consistent with the above analytical results.

## 5. Conclusions

This study systematically revealed the geochemical behavior, ecological hazard, and pollution sources of heavy metals and metalloids in topsoil and deep farmland soils of the Fenghuang polymetallic mining area through multi-dimensional analysis and PCA-PMF coupled source identification. Mining-derived anthropogenic inputs primarily drive Cd and Hg accumulation in surface soils, with evident vertical infiltration of contaminants into deep soil layers, which constitute the dominant ecological risk of the region. The four pollution sources quantified in this work match local mining, agricultural, traffic, and natural geological backgrounds well, and the reliability of the combined receptor model has been fully verified. Our findings deliver targeted theoretical support for farmland pollution remediation and safe agricultural production in western Hunan, and the summarized migration and source differentiation mechanisms are also applicable to analogous polymetallic mining–agricultural zones across Southeast Asia, South America, and Africa.

For future research, long-term in situ monitoring should be carried out to track the continuous vertical migration of heavy metals under seasonal rainfall and farming activities. Field passivation remediation trials targeting Cd and Hg will help develop practical risk control technologies. Moreover, combined hydrological and geochemical models can be adopted to simulate element transport at a larger watershed scale. Cross-regional comparative investigations of multi-metal mining areas are also recommended to form universal pollution prevention frameworks for global mining-affected farmlands.

## Figures and Tables

**Table 1:** Analysis methods and detection limits of various elements in soil samples.

Element	Measurement Method	Detection Limit	Element	Measurement Method	Detection Limit
As	AFS	0.20	Pb	ICP-MS	1.00
Hg	AFS	0.0005	Cu	ICP-MS	0.1
Cr	XRF	1.50	Zn	ICP-MS	1.00
Ni	ICP-AES	0.2	Cd	ICP-MS	0.02

Note: The detection limits for As, Cd, Cr, Cu, Hg, Pb, Ni, and Zn are in mg·kg^−1^.

**Table 2:** Criterion of ecological risk grade and metals and metalloids toxicity coefficient.

$E_{r}^{\;i}$ Category		RI Category	
$E_{r}^{\;i}$	Description	RI	Description
$E_{r}^{\;i}$ < 40	Low risk	RI < 150	Low risk
40 ≤ $E_{r}^{\;i}$ < 80	Moderate risk	150 ≤ RI < 300	Moderate risk
80 ≤ $E_{r}^{\;i}$ < 160	High risk	300 ≤ RI < 600	High risk
160 ≤ $E_{r}^{\;i}$ < 320	Ultra high risk	600 ≤ RI < 1200	Ultra high risk
$E_{r}^{\;i}$ ≥ 320	Extreme risk	RI ≥ 1200	Extreme risk

**Table 3:** Statistical characterization of heavy metal concentrations in topsoils (0–20 cm) of the study area.

Element	Data Description/mg·kg^−1^						
	Average	Median	Minimum	Maximum	Standard Deviation	Coefficient of Variation	Hunan Background Value
pH	6.17	6.06	4.05	7.99	0.83	13.46	—
Cr	75.22	71.6	47.1	178	14.71	19.56	71.4
Cd	0.47	0.38	0.11	2.65	0.3	64.59	0.13
Hg	0.42	0.2	0.04	8.64	1	239.21	0.12
As	14.95	12.8	1.61	58.4	8.77	58.66	15.7
Cu	32.84	31	18.7	75.3	8.78	26.75	27.3
Pb	42.15	35.1	18.8	162	20.78	49.3	29.7
Zn	103.14	93	55.5	325	35.7	34.61	94.4
Ni	36.02	35.35	18.8	75.7	8.06	22.37	31

Note: The soil element background values of Hunan Province are derived from the monograph Background Values of Soil Elements in China, published by China Environmental Science Press, Beijing, 1990.

**Table 4:** Statistical characterization of heavy metal concentrations in deep soils (150–180 cm) of the study area.

Element	Data Description/mg·kg^−1^						
	Average	Median	Minimum	Maximum	Standard Deviation	Coefficient of Variation	Hunan Background Value
pH	6.77	6.89	4.86	8.23	0.68	10.09	—
Cr	78.30	75.00	55.60	150.00	15.65	19.99	71.40
Cd	0.33	0.29	0.09	1.05	0.17	51.52	0.13
Hg	0.29	0.18	0.03	2.41	0.39	134.48	0.12
As	19.99	17.20	1.89	59.40	11.75	58.78	15.70
Cu	36.22	32.30	20.00	80.00	11.95	32.99	27.30
Pb	43.07	33.90	16.90	206.00	26.99	62.67	29.70
Zn	108.66	91.10	52.10	350.00	47.90	44.08	94.40
Ni	42.32	39.70	23.60	104.00	13.27	31.36	31.00

Note: The soil element background values of Hunan Province are derived from the monograph Background Values of Soil Elements in China, published by China Environmental Science Press, Beijing, 1990.

**Table 5:** Proportions of sites with different pollution levels in topsoil (0–20 cm) and deep soils (150–180 cm).

**Elements** **(topsoil)**	**Pollution index**	**Samples/%**	**Slight**	**Moderate**	**Severe**
		**Safe**			
Cd	P_i_	46.48	42.97	6.84	3.71
Hg	P_i_	28.18	32.88	17.22	21.72
As	P_i_	51.95	35.94	10.94	1.17
Cu	P_i_	56.16	42.66	1.17	0.00
Pb	P_i_	59.10	35.81	3.13	1.96
Cr	P_i_	69.34	30.27	0.39	0.00
Zn	P_i_	46.08	49.02	4.71	0.20
Ni	P_i_	24.02	75.20	0.78	0.00
	P_N_	21.88	46.48	14.65	16.99
**Elements** **(deep soil)**	**Pollution index**	**Samples/%**	**Slight**	**Moderate**	**Severe**
		**Safe**			
Cd	P_i_	6.12	48.98	27.21	17.69
Hg	P_i_	8.16	32.65	22.45	36.73
As	P_i_	29.25	40.82	21.09	8.84
Cu	P_i_	22.30	70.27	7.43	0.00
Pb	P_i_	34.01	47.62	11.56	6.80
Cr	P_i_	61.90	38.10	0.00	0.00
Zn	P_i_	25.85	61.90	9.52	2.72
Ni	P_i_	12.16	77.70	9.46	0.68
	P_N_	1.35	41.89	25	31.76

**Table 6:** Proportion of sites at different potential ecological risk levels.

Ecological Risk Level	Low Risk	Moderate Risk	High Risk	Ultra High Risk	Extreme Risk	Total
Number of Sampling Points	299	145	47	11	10	512
RI	58.40%	28.32%	9.18%	2.15%	1.95%	100%

**Table 7:** Metals and metalloid factor loadings in topsoil and deep soil.

Element	Topsoil			Deep Soil	
	Component 1	Component 2	Component 3	Component 1	Component 2
Ni	0.85	−0.34	−0.01	0.79	−0.31
Zn	0.83	0.47	−0.06	0.73	0.62
As	0.78	0.16	0.02	0.78	−0.38
Cd	0.77	−0.02	−0.01	0.50	0.27
Cr	0.76	−0.46	−0.09	0.64	−0.38
Pb	0.71	0.62	−0.04	0.53	0.79
Cu	0.71	−0.43	0.07	0.86	−0.30
Hg	0.09	0.03	0.99	0.45	0.08
Variance Contribution Rate %	52.50	14.11	12.55	45.76	19.63
Cumulative Contribution Rate %	52.50	66.61	79.16	45.76	65.39

## Data Availability

The raw data supporting the conclusions of this article will be made available by the authors on request.
